# A tangible method to assess native ferroptosis suppressor activity

**DOI:** 10.1016/j.crmeth.2024.100710

**Published:** 2024-02-24

**Authors:** Toshitaka Nakamura, Junya Ito, André Santos Dias Mourão, Adam Wahida, Kiyotaka Nakagawa, Eikan Mishima, Marcus Conrad

**Affiliations:** 1 Institute of Metabolism and Cell Death, Molecular Targets & Therapeutics Center, Helmholtz Zentrum München, 85764 Neuherberg, Bavaria, Germany; 2 Laboratory of Food Function Analysis, Tohoku University Graduate School of Agricultural Science, Sendai, Miyagi 980-8572, Japan; 3 Institute of Structural Biology, Molecular Targets & Therapeutics Center, Helmholtz Zentrum München, 85764 Neuherberg, Bavaria, Germany; 4 Division of Nephrology, Rheumatology and Endocrinology, Tohoku University Graduate School of Medicine, Sendai, Miyagi 980-8574, Japan

**Keywords:** GPX4, FSP1, lipid peroxidation, cell death, RSL3, selenocysteine, enzyme assay, affinity purification, pull-down assay, LC-MS/MS, drug discovery, biochemistry

## Abstract

Ferroptosis, a regulated cell death hallmarked by unrestrained lipid peroxidation, plays a pivotal role in the pathophysiology of various diseases, making it a promising therapeutic target. Glutathione peroxidase 4 (GPX4) prevents ferroptosis by reducing (phospho)lipid hydroperoxides, yet evaluation of its actual activity has remained arduous. Here, we present a tangible method using affinity-purified GPX4 to capture a snapshot of its native activity. Next to measuring GPX4 activity, this improved method allows for the investigation of mutational GPX4 activity, exemplified by the GPX4^U46C^ mutant lacking selenocysteine at its active site, as well as the evaluation of GPX4 inhibitors, such as RSL3, as a showcase. Furthermore, we apply this method to the second ferroptosis guardian, ferroptosis suppressor protein 1, to validate the newly identified ferroptosis inhibitor WIN62577. Together, these methods open up opportunities for evaluating alternative ferroptosis suppression mechanisms.

## Highlights

• A versatile method for assaying activity of the ferroptosis guardians GPX4 and FSP1
• The affinity-purified protein method captures a snapshot of native activity
• GPX4 prepared in this way responds to RSL3 inhibition
• Identification of WIN62577 as a human FSP1-specific inhibitor

## Motivation

To comprehend the regulatory mechanisms of ferroptosis, evaluating the enzymatic activity of GPX4, a key player in inhibiting lethal lipid peroxidation, is essential. Conventional GPX4 activity assays using whole-cell lysate have obstacles due to the presence of oxidoreductases contained in cell lysates. Additionally, challenges arise in producing recombinant GPX4 in bacteria, with the enzyme exhibiting unexpected resistance to well-known GPX4 inhibitors. To address these challenges, this study introduces a GPX4-specific activity assay employing affinity-purified GPX4 and purified lipid hydroperoxide. This method was extended to FSP1, opening avenues for investigating ferroptosis suppressor activities.

## Graphical abstract

**Figure:**
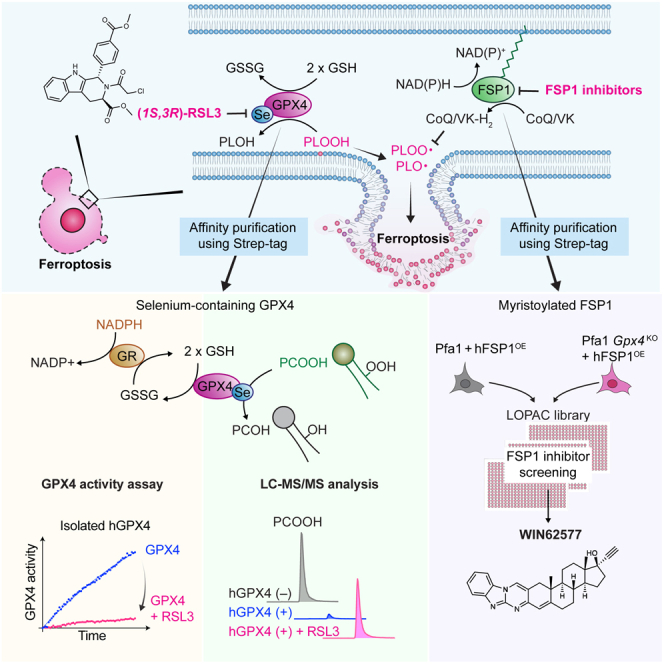

## Teaser

Lipid peroxidation triggers ferroptosis, a promising therapeutic target. Nakamura et al. present a tangible method that utilizes affinity-purified GPX4 and FSP1 to obtain a snapshot of native anti-ferroptotic activity. This assay opens up avenues for evaluating alternative ferroptosis regulatory mechanisms and for screening ferroptosis-inducing agents targeting key suppressors.

## Introduction

Ferroptosis is a regulated cell death hallmarked by excessive lipid oxidization^1^ with far-reaching implications for human disease, thus making it a promising therapeutic target.^2^^,^^3^^,^^4^ Consequently, ferroptosis is warranted as a promising therapeutic target for these diseases. Glutathione peroxidase 4 (GPX4) effectively impedes lethal lipid peroxidation by reducing (phospho)lipid hydroperoxide at the expense of glutathione (GSH). Thus, the proper evaluation of GPX4 activity is important for investigating molecular mechanisms of ferroptosis as well as developing drugs targeting ferroptosis. Notably, certain cancer cells exhibit intrinsic high vulnerability to GPX4 inhibition^5^^,^^6^ in synergism with inhibition of ferroptosis-suppressor protein 1 (FSP1),^7^^,^^8^^,^^9^^,^^10^^,^^11^^,^^12^ the second guardian of ferroptosis. It follows that the precise enzymatic evaluation of these systems is essential for investigating mechanisms of ferroptosis and for developing novel therapies; yet, conventional methods have several methodological obstacles for assessing inherent GPX4 activity.

First, the conventional assay is performed using whole-cell lysates in a cuvette,^13^ where GPX4 activity is monitored by measuring NADPH consumption, achieved by glutathione reductase (GR) coupled to the reduction of GSH upon reduction of the model substrate phosphatidylcholine hydroperoxide (PCOOH) by GPX4. Notably, these results might under- or overestimate the actual contribution of GPX4 due to the presence of numerous oxidoreductases in crude cell lysates. Second, GPX4 is a selenoprotein containing the 21^st^ amino acid selenocysteine (Sec) in its active site. Since bacteria lack the selenium incorporation machinery to decode the UGA codon in mammalian selenoprotein mRNAs including *GPX4*, this characteristic renders it inherently challenging to produce recombinant GPX4 in bacteria. Recently, heterologous expression and purification of Sec-containing GPX4 have been achieved by utilizing a modified bacterial strain.^14^ However, this strategy only yields a fraction of Sec-containing GPX4, whereby additional purification steps are required to obtain homogeneous Sec-GPX4.^14^ Moreover, this enzyme is unexpectedly resistant to the inhibitory properties of (1*S,*3*R*) RSL3 (RSL3),^15^ the *bona fide* GPX4 inhibitor.^16^^,^^17^ It is further reported that the cellular native GPX4 activity might be impacted by post-translational modifications (PTMs).^18^ To overcome these limitations, we developed a straightforward GPX4-specific activity assay using both the affinity-purified GPX4 from mammalian cells and purified lipid hydroperoxide. Isolated GPX4, in turn, can meet a need for a widely accessible method that accurately reflects GPX4 activity, evaluating the potential impact of PTMs and mutant variants as well as known and future inhibitors of GPX4. Furthermore, we apply this method to another ferroptosis guardian, FSP1, which breaks new ground for studying the ferroptosis suppression mechanism.

## Results

### Whole-cell lysate samples are not optimal for conventional GPX4 activity assay

**Figure 1:**
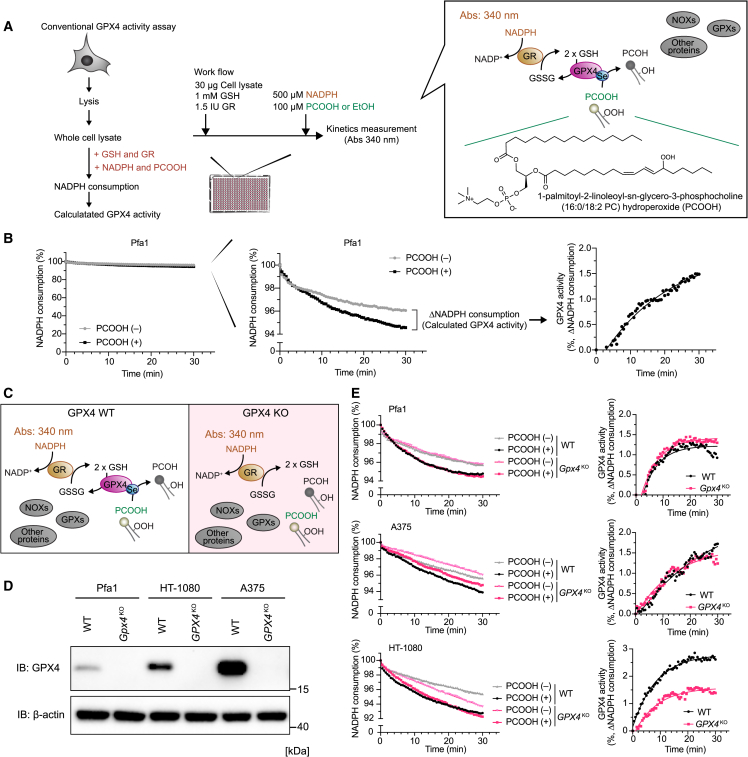
Whole-cell lysate samples are not optimal for conventional GPX4 activity assay (A) Schematic model of the conventional GPX4 activity assay. Cells are lysed, and then whole-cell lysates are incubated with glutathione (GSH), glutathione reductase (GR), NADPH, and purified 1-palmitoyl-2-linoleoyl-*sn*-glycero-3-phosphocholine (16:0/18:2 PC) hydroperoxide (phosphatidylcholine hydroperoxide [PCOOH]). NADPH consumption by GR coupled with GSH reduction is expected to be the same molar ratio as PCOOH reduction by GPX4. (B) Representative results of NADPH consumption using whole-cell lysates from 4-hydroxy-tamoxifen-inducible *Gpx4* knockout (KO) mouse embryonic fibroblasts and Pfa1 cells (left). Zoomed y axis of NADPH consumption (middle). GPX4 activity was calculated using NADPH consumption in the presence and absence of PCOOH (right). (C) Schematic model of the conventional GPX4 activity assay using wild-type (WT) and *GPX4*^KO^ cells. (D) Immunoblots of GPX4 in WT and GPX4-deficient Pfa1, HT-1080, and A375 cells. β-Actin is shown as a loading control. (E) Representative zoomed results of NADPH consumption using whole-cell lysates of WT and *GPX4*^KO^ cells (left). GPX4 activity was calculated using NADPH consumption in the presence and absence of PCOOH (right). Data represent the mean of 3 technical replicates from 1 out of 3 independent experiments (B and E).

To measure GPX4 activity, we initially performed a conventional GPX4 activity assay,^13^ monitoring GSH/GR-mediated NADPH consumption using a whole-cell lysate extracted from tamoxifen-inducible *Gpx4* knockout (KO) mouse embryonic fibroblasts (Pfa1 cells)^19^ and prepared pure PCOOH (1-palmitoyl-2-linoleoyl-*sn*-glycero-3-phosphocholine hydroperoxide [16:0/18:2-PCOOH])^20^ as the model substrate of GPX4 (Figure 1A). Since this conventional assay is typically performed in cuvettes,^21^ it was difficult to simultaneously track the kinetics in the presence and absence of PCOOH. Thus, to facilitate a more precise comparison of GPX4-specific activity, we developed a high-throughput format using a microplate (384- or 96-well plates).^22^ Although the addition of PCOOH is reduced by GPX4 and should lead to the increase in consumption of NADPH, the effect of PCOOH on NADPH consumption was very small in this conventional assay, consistent with observations in numerous previous studies^23^ (Figure 1B). To ascertain the specificity of this conventional method evaluating the GPX4 activity in whole-cell lysate samples, we performed the assay using crude cell lysates derived from wild-type (WT) cells and *GPX4*^KO^ mouse and human cells (Figures 1C and 1D). Notably, NADPH consumption occurred even in the absence of PCOOH, and the calculated GPX4 activity based on the NADPH consumption was not significantly different between cell lysates from the WT Pfa1 cells and that of *Gpx4*^KO^ cells, even when supplemented with PCOOH (Figure 1E). Similar results were obtained using this assay in cell lysates from WT and *GPX4*^KO^ human melanoma A375 cells (Figure 1E). In the case of human fibrosarcoma HT-1080 cells, the lysate from *GPX4*^KO^ cells exhibited slightly slower NADPH consumption than WT samples, suggesting a lower GPX4 activity in the *GPX4*^KO^ cell lysates. However, the difference in the calculated GPX4 activity was marginal and only differed by 1%–2% between WT and *GPX4*^KO^ cells, which is hardly suitable for making a strong conclusion on the actual activity of GPX4 in cells. The decrease of NADPH in the absence of PCOOH is likely attributed to GPX4-independent NADPH consumption and/or GSH consumption, mediated by NADPH oxidases and other GSH-consuming oxidoreductases present in whole-cell lysates. Therefore, whole-cell lysates were considered not to be the best starting material for conducting the GPX4-specific activity assay.

### GPX4 activity assay using selenium-containing isolated GPX4

**Figure 2:**
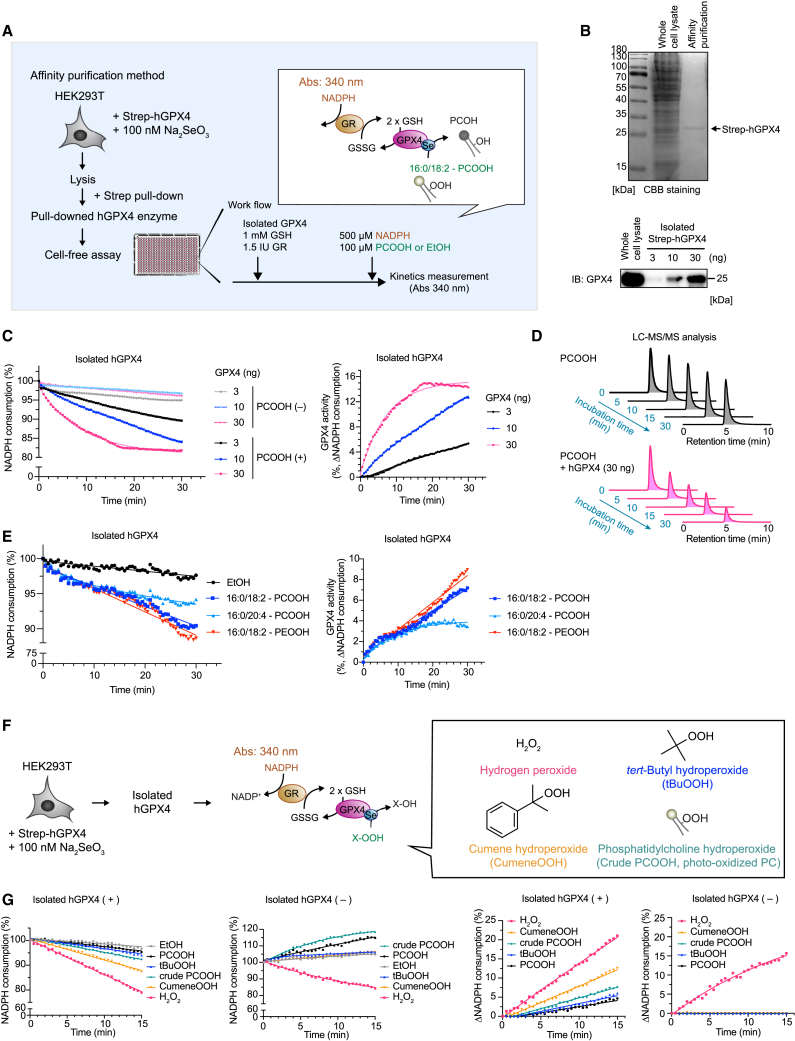
Method for assessing selenium-containing GPX4 activity upon affinity purification (A) Schematic model of the method. A plasmid encoding 2× Strep-tagged (Strep)-hGPX4 was transiently transfected into HEK293T cells. After 48–72 h of incubation in medium containing Na_2_SeO_3_ (100 nM), the collected cells were lysed, and Strep-hGPX4 was isolated by MagStrep beads. Isolated hGPX4, GSH (1 mM), and GR (1.5 IU) were added to the assay plate, and then NADPH (500 μM) and purified standard-grade PCOOH (100 μM) were simultaneously added immediately before the start of the kinetic assay (absorbance 340 nm). (B) Coomassie brilliant blue (CBB) staining image of whole-cell lysate of HEK293T cells expressing Strep-hGPX4 and affinity-purified samples (600 ng). An arrow indicates the band of Strep-hGPX4. Immunoblot analysis shows that the CBB-detected band corresponds to hGPX4. (C) Representative zoomed results of NADPH consumption using different concentrations of isolated hGPX4 (left). GPX4 activity is calculated based on NADPH consumption in the presence and absence of PCOOH (right). (D) Relative amount of remaining PCOOH analyzed by LC-MS/MS after incubation with and without 30 ng isolated hGPX4 for the indicated time at 37°C. Data represent the intensity of PCOOH. The same data as Figure 4G are shown from a single experiment. (E) Representative zoomed results of NADPH consumption using 16:0/18:2-PCOOH, 16:0/20:4-PCOOH, and 16:0/18:2-PEOOH as substrates of GPX4 (left). GPX4 activity is calculated based on NADPH consumption in the presence and absence of phospholipids hydroperoxides. (F) Schematic model using alternative substrates of GPX4. Isolated hGPX4 was incubated with GSH, GR, NADPH, and different peroxide substrates (i.e., H_2_O_2_, *tert*-butyl hydroperoxide [tBuOOH], cumene hydroperoxide [cumeneOOH], and crude PCOOH prepared by oxidizing soybean phosphatidylcholine using a photosensitizer) as well as purified standard-grade PCOOH. (G) Representative zoomed results of NADPH consumption (left) and calculated delta NADPH consumption by comparing the values in the presence or absence of isolated hGPX4 (right) using different substrates. Data represent the mean of 3 technical replicates from one out of 2 (E) or 3 (C and G) independent experiments.

For the determination of the specific activity of GPX4, the availability of a purified Sec-containing GPX4 enzyme is essential, as its variant with a targeted Sec-to-cysteine version is not stable outside cells and is immediately overoxidized when exposed to peroxidatic substrates.^21^ However, the production of recombinant selenium-containing GPX4 in bacteria can be challenging without the use of a specialized strain and purification systems.^14^ In contrast, GPX4 directly isolated from mammalian cells may have clear advantages because it also allows us to monitor the native GPX4 activity that may be affected by potential PTMs.^18^ To this end, we set out to isolate GPX4 directly from mammalian HEK293T cells transiently overexpressing human GPX4 (hGPX4) furnished with an N-terminal 2× Strep Tag II by affinity purification (Figure 2A and S1). To enhance expression of selenoproteins, sodium selenite was supplemented to the culture medium concomitantly with transfection. Two to three days following transfection, transfected HEK293T cells were harvested and lysed. The lysate was centrifuged, and the supernatant was subjected to incubation with magnetic beads to isolate Strep-tagged hGPX4 (Strep-hGPX4), followed by elution with the elution buffer. Coomassie brilliant blue (CBB) staining showed a single band corresponding to tagged GPX4, as also confirmed by immunoblotting (Figure 2B).

To investigate the applicability of isolated GPX4 in the GPX4 activity assay, various amounts of isolated hGPX4 enzyme were added to the assay buffer, and then NADPH consumption was monitored over time. In comparison to the assay using whole-cell lysate, minimal NADPH consumption was evident in the absence of PCOOH during the assay using isolated GPX4 (Figure 2C). By contrast, in the presence of PCOOH, a clear consumption of NADPH was observed, correlating with the amounts of GPX4 present in the assay. This resulted in a marked improvement in the resolution of GPX4-dependent NAPDH consumption and allowed us to actually calculate GPX4 activity (2%–15% as the calculated GPX4 activity) (Figure 2C). To provide further proof that PCOOH is indeed enzymatically reduced by isolated hGPX4, we assessed the amount of PCOOH before and after incubation with isolated hGPX4 in a time-dependent manner using liquid chromatography-tandem mass spectrometry (LC-MS/MS). This analysis clearly validated GPX4-dependent reduction of PCOOH (Figure 2D). In addition to PCOOH (16:0/18:2-PCOOH), we included other phospholipid hydroperoxides, i.e., phosphatidylethanolamine (PE) hydroperoxide (16:0/18:2-PEOOH) and arachidonic acid-containing PCOOH (16:0/20:4-PCOOH), since PE hydroperoxide containing arachidonic acid have been repeatedly reported to play a pivotal role in ferroptosis induction.^24^^,^^25^ Our assay system demonstrates that these phospholipids hydroperoxides also serve as good model substrates of GPX4 (Figure 2E). Besides, we included other hydroperoxides, such as hydrogen peroxide (H_2_O_2_), *tert*-butyl hydroperoxide, and cumene hydroperoxide, all of which are considered to be substrates of GPX4^26^ (Figure 2F). All hydroperoxides except for H_2_O_2_ afforded the GPX4-dependent NADPH consumption in the assay system (Figure 2G) to the same extent as previous studies using recombinant selenium-containing GPX4 produced in bacteria.^26^ Yet, H_2_O_2_ alone consumed NADPH even in the absence of GPX4 (Figure 2G, left). indicating that H_2_O_2_ is not a suitable substrate for this method. Furthermore, to enhance the accessibility of this assay, we also tested crude PCOOH generated by photo-oxidization of soybean phosphatidylcholine as a substrate of GPX4 instead of pure PCOOH. Crude PCOOH also exhibited comparable PCOOH/GPX4-dependent NADPH consumption like pure PCOOH (Figures 2C and 2G). Therefore, this method can reliably assess the specific activity of the isolated hGPX4 enzyme.

### Evaluation of the GPX4 activity of GPX4^U46C^ mutant lacking catalytically active Sec

**Figure 3:**
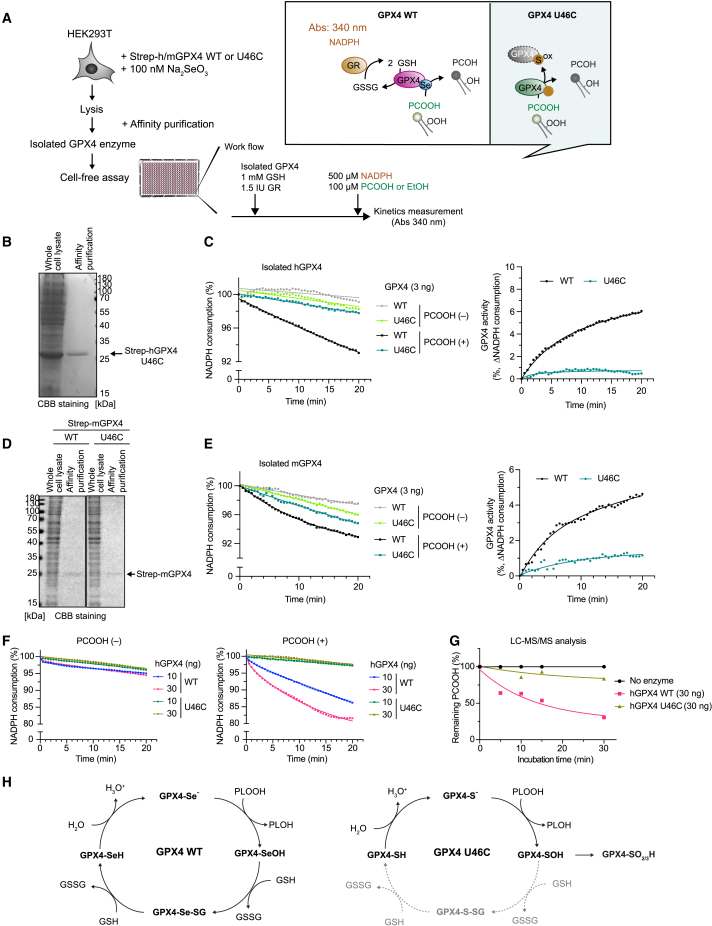
Validation of the GPX4 activity assay using a GPX4 variant carrying a selenocysteine-to-cysteine substitution (A) Schematic model for evaluating GPX4 activity of hGPX4 and hGPX4^U46C^ mutant. HEK293T cells transiently expressing Strep-hGPX4 or hGPX4^U46C^ were lysed, and Strep-hGPX4 was isolated by affinity purification. Isolated hGPX4 was used for the GPX4 activity assay as shown in Figure 2A. (B) CBB staining image of whole-cell lysates of HEK293T cells expressing hGPX4^U46C^ and the affinity-purified sample. The arrow indicates Strep-hGPX4^U46C^. (C) Representative zoomed results of NADPH consumption and calculated GPX4 activity using isolated hGPX4 and hGPX4^U46C^. (D) CBB staining image of whole-cell lysates of HEK293T cells expressing mGPX4 and mGPX4^U46C^ and the affinity-purified sample. The arrow indicates Strep-mGPX4 and mGPX4^U46C^. (E) Representative zoomed results of NADPH consumption and calculated GPX4 activity using isolated mGPX4^WT^ and mGPX4^U46C^. (F) Representative zoomed results of NADPH consumption using different concentrations of isolated hGPX4^WT^ and hGPX4^U46C^ in the presence and absence of PCOOH. (G) Relative amount of remaining PCOOH analyzed by LC-MS/MS after incubation with and without 30 ng of GPX4^WT^ and GPX4^U46C^ at 37°C for the indicated time. The amount in the PCOOH sample without enzyme is taken as 100%. The same data as in Figure 2D is shown from a single experiment. Data represent the mean of 3 technical replicates from 1 out of 3 independent experiments (C, E, and F). (H) Schematic model of the enzymatic steps for the reduction of (phospho)lipid peroxides by GPX4. Selenium (Se) in the form of the 21^st^ proteinogenic amino acid selenocysteine is oxidized by phospholipid hydroperoxides (PLOOH), yielding PLOH and selenenic acid. Selenenic acid is regenerated in two consecutive steps by 2 mol GSH (left). In contrast, when selenocysteine is replaced with cysteine in the GPX4^U46C^ mutant, the thiolate in the active-site GPX4 is highly prone to undergo peroxide-induced irreversible overoxidation by forming sulfinic and sulfonic acids, which cannot be regenerated by GSH in this assay context (right).

To address the versatility of this method for mutational analysis of GPX4, we examined the GPX4 activity of the GPX4^U46C^ mutant, in which Sec (U46) in its active site is substituted by cysteine, just as an example^21^ (Figure 3A). Like hGPX4, hGPX4^U46C^ was isolated by affinity purification from the lysate of the HEK293T cells transiently overexpressing Strep-hGPX4^U46C^. CBB staining showed a single band corresponding to hGPX4^U46C^ (Figure 3B). In the GPX4 activity assay, the mutant enzyme showed minimal PCOOH-dependent NADPH consumption, indicating the decreased GPX4 enzyme activity of the U46C mutant (Figure 3C). Next, we tested the GPX4 activity of murine GPX4 (mGPX4) and mGPX4^U46C^ (Figure 3D). Like the results obtained for hGPX4^U46C^, mGPX4^U46C^ showed strongly decreased GPX4 activity, as evidenced by the lower PCOOH-dependent NADPH consumption (Figure 3E). While mGPX4 showed dose-dependent GPX4 activity, its hGPX4^U46C^ mutant showed very little GPX4 activity, even when using higher amounts of the enzyme (Figure 3F). To validate that the GPX4 activity assay indeed reflects phospholipid hydroperoxidase activity, we directly measured residual amounts of PCOOH after incubation with an hGPX4 or hGPX4^U46C^ enzyme using LC-MS/MS. In fact, hGPX4 effectively reduced PCOOH, as illustrated in Figure 2D, while the hGPX4^U46C^ mutant exhibited a strongly decreased reduction of PCOOH (Figure 3G). During catalysis of WT GPX4, the selenolate of GPX4 (GPX4-Se^−^) reacts first with a hydroperoxide, such as PCOOH, yielding alcohol (i.e., PCOH) and selenenic acid (GPX4-SeOH), the oxidized form of GPX4 (Figure 3H, left). Subsequently, selenenic acid is regenerated to its ground state in two steps, by reacting first with one molecule of GSH, forming a mixed selenadisulfide (GPX4-Se-SG), and then with another molecule of GSH, forming the selenthiol form of GPX4 (GPX4-SeH) and di-glutathione (GSSG) (Figure 3H, left). In contrast, in the GPX4^U46C^ mutant, the cysteine in the active site is prone to become irreversibly oxidized, forming sulfinic (-SO_2_H) and sulfonic acid (-SO_3_H) following its reaction with PLOOH. These oxidized forms cannot be regenerated to the fully reduced thiol form by GSH, explaining the lack of NADPH consumption in the GPX4 activity assay (Figure 3H, right).^27^ Taken together, the results shown by this method are consistent with the proposed enzymatic mechanism of the GPX4^U46C^ mutant, indicating that this method can also be easily applied when studying distinct mutant variants of GPX4.

### Evaluating the inhibitory effect of RSL3 on GPX4 activity

**Figure 4:**
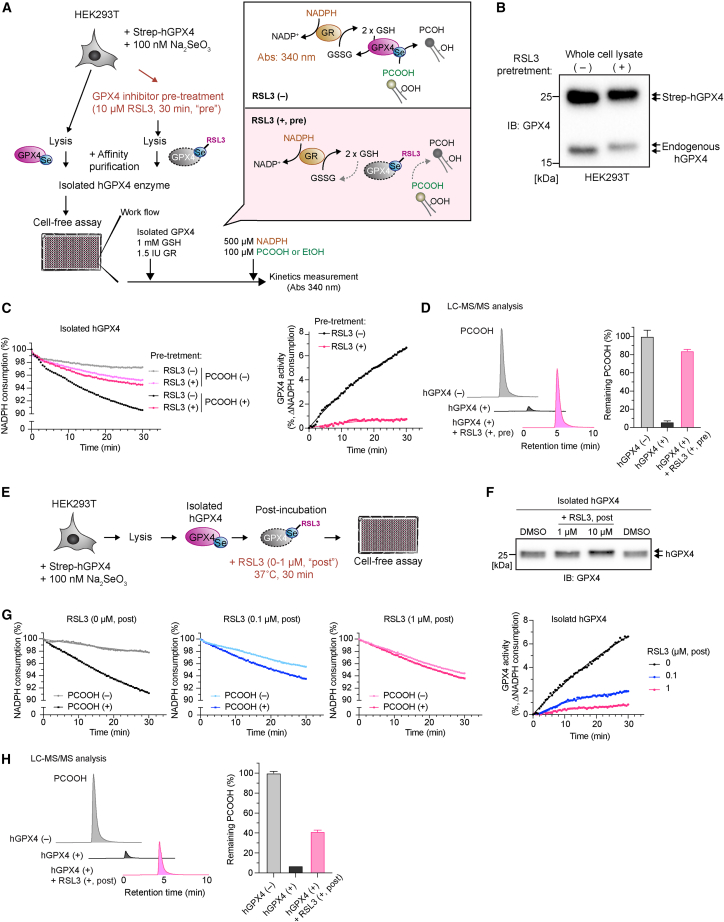
Validation of the GPX4 activity assay using the GPX4 inhibitor RSL3 (A) Schematic model for evaluating the inhibitory effect of RSL3 using RSL3-pretreated cell samples. HEK293T cells transiently expressing Strep-hGPX4 were pretreated with RSL3 (10 μM) for 30 min. Strep-hGPX4 isolated from the lysate of the RSL3-pretreated cells was used for the GPX4 activity assay. (B) GPX4 immunoblot analysis of lysates collected from HEK293T cells pretreated with RSL3 (10 μM) for 30 min. The upper bands represent the forced expression of Strep-hGPX4, and the lower bands represent endogenous GPX4. RSL3 pretreatment causes the band shift in both GPX4. (C) Representative zoomed results of NADPH consumption using isolated hGPX4 pretreated (+, pre) or untreated (−) with RSL3 (top). GPX4 activity was calculated based on NADPH consumption in the presence and absence of PCOOH (bottom). (D) Relative amount of remaining PCOOH analyzed by LC-MS/MS after incubation (37°C, 30 min) with and without hGPX4 (30 ng) and hGPX4 isolated from RSL3 (10 μM)-pretreated cells (+, pre). The amount in the PCOOH sample without enzyme is taken as 100%. (E) Schematic model for evaluating the inhibitory effect of RSL3 using Strep-hGPX4 incubated with RSL3 after affinity purification (post). Isolated hGPX4 was incubated with RSL3 (0, 0.1, and 1 μM) at 37°C for 30 min, and then GPX4 activity was measured. The reaction was initiated upon incubation with GSH, GR, NADPH, and purified PCOOH. (F) Immunoblotting of isolated hGPX4 after treatment with 0, 1, and 10 μM RSL3 for 30 min. (G) Representative zoomed results of NADPH consumption and calculated GPX4 activity using isolated hGPX4 treated with different concentrations of RSL3 (+, post). (H) Relative amount of remaining PCOOH analyzed by LC-MS/MS after incubation (37°C, 30 min) with and without hGPX4 (30 ng) and hGPX4 incubated with RSL3 after affinity purification (1 μM; +, post). The amount in the PCOOH sample without enzyme is taken as 100%. Data represent mean + SD of 3 technical replicates from a single (D) or 2 independent (H) experiments. Data represent the mean of 3 technical replicates from 1 out of 3 independent experiments (C and G).

Next, we asked whether this method is suitable for evaluating the impact of GPX4 inhibitors. To this end, we treated HEK293T cells overexpressing Strep-hGPX4 with the most studied GPX4 inhibitor, RSL3 (Figure 4A).^16^^,^^17^ Due to its chloroacetamide warhead, RSL3 is known to irreversibly inhibit GPX4 by covalently binding to the Sec (U46) residue (Figure 4A).^21^^,^^28^ After subjecting cells to a 30 min pretreatment with RSL3, cells were harvested, and Strep-hGPX4 was isolated from lysates (Figure 4A). Immunoblot analysis demonstrated the band shift of Strep-hGPX4 by RSL3 pretreatment, indicating the covalent binding of RSL3 to GPX4 as reported earlier (Figure 4B).^29^ Notably, the NADPH consumption assay revealed that GPX4 isolated from RSL3-pretreated cells exhibited far less GPX4 activity compared to that from RSL3-untreated cells (Figure 4C). Besides, LC-MS/MS analysis confirmed the complete inhibition of GPX4 activity in Strep-hGPX4 in response to RSL3 pretreatment (Figure 4D). These data clearly indicate that RSL3 inhibits GPX4 activity at least in the cellular context.

We next examined whether RSL3 has a direct inhibitory effect on isolated hGPX4. After isolating Strep-hGPX4 from cell lysates, Strep-hGPX4 was incubated with RSL3 for 30 min and analyzed by immunoblotting and GPX4 activity assay (Figure 4E). Of note, after RSL3 treatment of the isolated enzyme, the band shift of GPX4 in immunoblotting was noticeable in a dose-dependent manner (Figure 4F), mirroring the pattern observed in the cells pretreated with RSL3 (Figure 4B). These data strongly suggest that RSL3 can indeed alkylate GPX4 even in a cell-free condition. We validated these findings by showing that Strep-hGPX4 treated with RSL3 after isolation had diminished GPX4 activity (Figure 4G). LC-MS/MS analysis evaluating PCOOH reduction via GPX4 confirmed that RSL3 can readily inhibit GPX4 in a cell-free context (Figure 4H). These findings thus reveal that RSL3 effectively inhibits GPX4 activity both in a cellular context and in a cell-free condition, providing proof of concept that the isolated GPX4 is suitable for assessing the inhibitory effect of GPX4 inhibitors.

### Evaluating the inhibitory effect of FSP1 inhibitors with isolated FSP1

**Figure 5:**
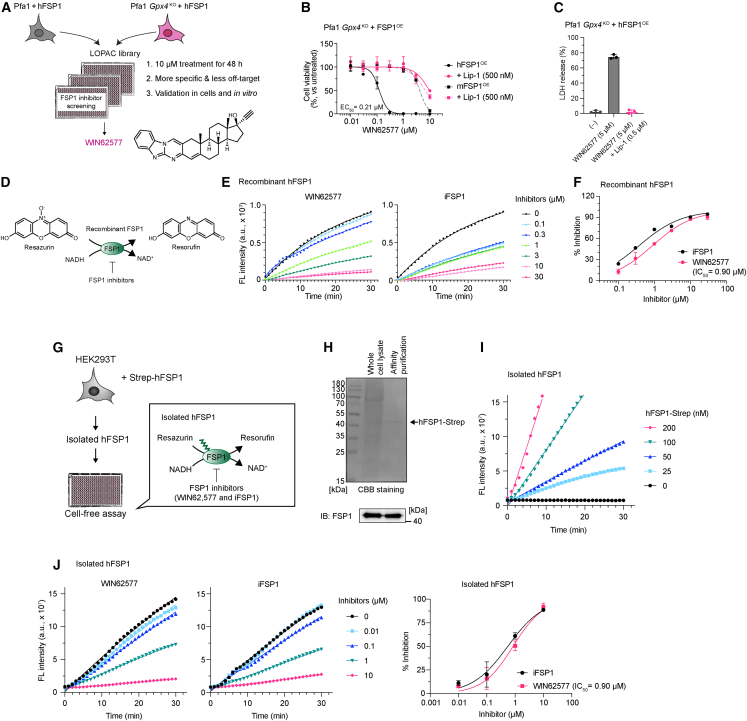
WIN62577 is a potent hFSP1 inhibitor (A) Schematic screening model for identifying a FSP1 inhibitor using the LOPAC library. The structure of WIN62577 is shown. (B) Cell viability of *Gpx4*^KO^ Pfa1 cells stably overexpressing hFSP1 and mouse FSP1 (hFSP1^OE^ and mFSP1^OE^, respectively) treated with WIN62577 alone or in combination with the ferroptosis inhibitor liproxstatin-1 (Lip-1, 0.5 μM) for 24 h. (C) Lactate dehydrogenase (LDH) release determined after treating *Gpx4*^KO^ Pfa1 cells overexpressing hFSP1 with untreated, WIN62577 (5 μM), or WIN62577 + Lip-1 (0.5 μM) for 24 h. (D) Schematic representation of the FSP1 enzyme activity assay using recombinant hFSP1 and resazurin as a substrate. (E) Representative reaction curves of FSP1 activity assay using recombinant hFSP1. FSP1 inhibitory activity of WIN62577 and iFSP1 was assessed by fluorescent (FL) intensity of reduced form of resazurin. (F) Representative dose-response curves showing the FSP1 inhibitory activity (%) of WIN62577 and iFSP1 on recombinant hFSP1. (G) Schematic representation of the FSP1 enzyme activity assay using isolated hFSP1 and resazurin. (H) CBB staining image of whole-cell lysate of HEK293T cells expressing Strep-hFSP1 and affinity-purified samples. An arrow indicates the band of hFSP1-Strep. Immunoblot analysis shows that the CBB-detected band corresponds to hFSP1. (I) Representative reaction curves of FSP1 activity assay using the different concentrations of isolated hFSP1. (J) (Left) Representative reaction curves of FSP1 activity assay using isolated hFSP1. WIN62577 and iFSP1 toward isolated hFSP1 (50 nM) were assessed by FL intensity of reduced form of resazurin. (Right) Representative dose-response curves for the effect of iFSP1 and WIN62577 on hFSP1 activity using isolated hFSP1 protein. Data represent the mean ± SD (B, C, and F) or mean (E) of 3 wells of a 96-well plate from 1 of 2 independent experiments. Data represent a single well of a 384-well plate from 3 independent experiments (I). Data represent the mean (J, left) or mean ± SD (J, right) of 3 wells of a 384-well plate from 1 of 2 independent experiments (J, left).

In addition to GPX4 inhibitors, the development of FSP1 inhibitors is also warranted as a promising approach for treating ferroptosis-vulnerable tumors. Recently, there has been a resurgence of interest in the repurposing of existing drugs, as it is a safe strategy that allows for their immediate and straightforward use.^30^ To this end, the Library of Pharmacologically Active Compounds (LOPAC1280) library, which contains 1,280 pharmacologically active compounds, was screened using Pfa1 *Gpx4*^KO^ cells overexpressing hFSP1 cells, whose viability solely depends on FSP1 activity (Figure 5A).^8^^,^^9^^,^^11^ This screening revealed that WIN62577, which is regarded as an NK1 tachykinin receptor antagonist,^31^ is a yet-unrecognized FSP1 inhibitor. WIN62577 induced ferroptosis in Pfa1 *Gpx4*^KO^ cells overexpressing hFSP1 (Figures 5B and 5C) and directly inhibits FSP1 activity of the recombinant hFSP1 enzyme with nearly the same potency as the first described specific hFSP1 inhibitor, iFSP1 (Figures 5D–5F).^9^^,^^24^

To demonstrate that the method used in the GPX4 activity assay can be applied for ferroptosis players besides GPX4, we generated cells expressing 2× Strep-hFSP1. Upon affinity purification, isolated hFSP1 was subsequently subjected to FSP1 enzyme assays in the presence or absence of WIN62577 or iFSP1 (Figure 5G). The advantage of the affinity-based isolation over recombinant enzyme is that it allows us to isolate and test native protein preserving potential PTMs, which is particularly relevant for FSP1, as it contains an N-terminal myristoylation.^11^^,^^12^ Accordingly, we successfully isolated tagged FSP1, maintaining potent enzyme activity (Figures 5H and 5I). The inhibitory effects of WIN62577 and iFSP1 against hFSP1 were subsequently investigated in the resazurin-based activity assay using isolated Strep-hFSP1. The kinetics and IC_50_ values of the inhibitory effect of both FSP1 inhibitors were comparable to those using recombinant hFSP1 proteins, showing that our method can be readily applied to enzymes other than GPX4.

## Discussion

We introduced here a simple and straightforward method that enables the rapid determination of native enzyme activities and related inhibitors. This method is particularly useful when assaying enzymes inherently difficult to be expressed in bacteria or other state-of-the-art heterologous expression systems.

We developed this approach for the key ferroptosis regulator GPX4, as it is one of 25 Sec-containing proteins in mammals that, due to an in-frame UGA codon, cannot be readily expressed in bacteria because selenoprotein biosynthetic machineries between bacteria and mammals vary substantially. Yet, the expression of mammalian selenoproteins was recently accomplished using the RF1-depleted *E. coli* strain C321.ΔA,^14^ yielding Sec-containing enzymes including GPX4. Although this bacterial strain produces Sec-containing GPX4, only a fraction (10%–20%) of the recombinant enzyme actually contained Sec.^14^ Moreover, this enzyme was not inhibitable by RSL3, a canonical GPX4 inhibitor widely used in ferroptosis research,^17^ questioning whether RSL3 is a direct inhibitor of the active-site Sec of GPX4. In contrast to these studies, utilizing isolated GPX4 and pure PCOOH as substrates, we not only successfully assessed GPX4-specific activity from mammalian cells but also showed that RSL3 is indeed a direct GPX4 inhibitor. Specifically, we found that RSL3 inhibited GPX4 not only when cells were treated with RSL3 prior to cell lysis but also when isolated GPX4 enzyme was exposed to RSL3 in the test tube. This implies that during heterologous expression and/or multi-step purification of recombinant enzyme, GPX4 must undergo some modifications in its active-site Sec. This hypothesis is supported by a previous study,^15^ in which the band of recombinant GPX4 detected in immunoblot analysis was indeed different from that of native cellular GPX4. These differences, which might be caused by oxidation and/or other PTMs, might impede the alkylating activity of RSL3. Besides being able to interrogate PTMs and potential binding partners in contrast to using recombinant GPX4 isolated from bacteria or other expression systems, our assay system offers several other advantages as it is (1) achievable with standard equipment, (2) rapid and convenient, (3) versatile and not limited to GPX4, and (4) scalable so that an endogenous enzyme can be used for even setting up (high-throughput) screening approaches.

### Limitations of the study

Since affinity-purified GPX4 and FSP1 are not entirely pure fractions, the possibility remains that these proteins were co-isolated with interacting proteins. Notably, the conformational stabilization of GPX4 by the chaperones may be necessary for RSL3-mediated GPX4 inhibition, as previously proposed.^32^ Indeed, given that GPX4 inhibition by RSL3 was more potent under intracellular conditions than cell-free reactions (Figures 4D and 4H), yet-unrecognized cofactors or varying redox conditions or the membrane environment might be required for an effective inhibition of GPX4 by RSL3 and other GPX4 inhibitors.

## STAR★Methods

### Key resources table

**Table:** 

REAGENT or RESOURCE	SOURCE	IDENTIFIER
**Antibodies**		

Rabbit monoclonal anti-GPX4 (1:1000)	Abcam	Cat# ab125066; RRID: AB_10973901
Mouse monoclonal anti-FSP1 (AMID, 1:1000)	Santa Cruz	Cat# sc-377120; RRID: AB_2893240
Mouse monoclonal anti-β-actin-HRP (1:50,000)	Sigma Aldrich	Cat# A3854; RRID: AB_262011
Horse anti-mouse-IgG-HRP (1:3000)	Cell Signaling	Cat# 7076S
Goat anti-rabbit-IgG-HRP (1:3000)	Cell Signaling	Cat# 7074S

**Chemicals, peptides, and recombinant proteins**		

(1*S,*3*R*)-RSL3 (RSL3)	Cayman	Cat# Cay19288
Liproxstatin-1 (Lip-1)	Selleckchem	Cat# S7699
iFSP1	Cayman	Cat# Cay29483
β-nicotinamide adenine dinucleotide 2′-phosphate reduced tetrasodium salt hydrate (NADPH)	Sigma Aldrich	Cat#10107824001
β-Nicotinamide adenine dinucleotide (NADH)	Sigma Aldrich	Cat# N8129
WIN62577	Sigma Aldrich	Cat# W104
LOPAC®1280 (LOPAC library)	Sigma Aldrich	Cat# LO1280
Resazurin sodium salt	Sigma Aldrich	Cat# R7017
Glutathione (GSH)	Sigma Aldrich	Cat# G4251
Glutathione reductase (GR)	Sigma Aldrich	Cat# G3664
Soybean phosphatidylcholine (PC)	Sigma Aldrich	Cat# P7443
Sodium selenite (Na_2_SeO_3_)	Sigma Aldrich	Cat# S5261
Hydrogen peroxide (H_2_O_2_)	Sigma Aldrich	Cat# H1009
Cumene hydroperoxide (cumeneOOH)	Sigma Aldrich	Cat# 247501
*tert-butyl* hydroperoxide (tBuOOH)	Sigma Aldrich	Cat# 458139
Rose bengal	Wako	Cat# 184-00272
MagStrep XT beads	IBA Lifesciences	Cat# 2-4090-002
PEI MAX	Polysciences	Cat# 24765
PCOOH (1-palmitoyl-2-hydroperoxyoctadecadienoyl-phosphatidylcholine, 16:0/18:2-PCOOH)	Ito et al.^20^	N/A
1-palmitoyl-2-arachidonoyl-*sn*-glycero-3-phosphocholine (16:0/20:4 PC)	Avanti	Cat# 850459C
16:0/20:4-PCOOH	This paper	N/A
1-stearoyl-2-linoleoyl-*sn*-glycero-3-phosphoethanolamine (16:0/18:2 PE)	Avanti	Cat# 850802C
16:0/18:2-PEOOH	This paper	N/A
Coomassie Brilliant Blue G-250	Sigma Aldrich	Cat# 115444002
In Fusion SNAP assembly	Takara Bio	Cat# 638948
Recombinant human FSP1	Doll et al.^11^	N/A

**Critical commercial assays**		

Cytotoxicity Detection kit (LDH assay kit)	Roche	Cat# 11644793001

**Experimental models: Cell lines**		

Human: HEK293T	ATCC	CRL-3216
Human: A375	ATCC	CRL-1619
Human: HT-1080	ATCC	CCL-121
Human: A375 *GPX4*^KO^	Mishima et al.^10^	N/A
Human: HT-1080 *GPX4*^KO^	Mishima et al.^7^	N/A
Mouse: 4-OH-tamoxifen (Tam)-inducible *Gpx4*^*−/−*^ murine immortalized fibroblasts (Pfa1 cells)	Seiler et al.^19^	N/A
Mouse: Pfa1 *Gpx4*^KO^	This paper	N/A
Mouse: Pfa1 *Gpx4*^KO^ + hFSP1^OE^	Nakamura et al.^8^	N/A
Mouse: Pfa1 *Gpx4*^KO^ + mFSP1^OE^	Nakamura et al.^8^	N/A

**Oligonucleotides**		

Primer for hGPX4 U46C-forward: 5′-aacgtggcctcccagtgCggcaagaccgaagta-3′	This paper	N/A
Primer for hGPX4 U46C-forward: 5′-tacttcggtcttgccGcactgggaggccacgtt-3′	This paper	N/A

**Recombinant DNA**		

Plasmid:141-Strep-hGPX4 (NM_001367832.1)-IRES-puro	This paper	N/A
Plasmid:141-Strep-hGPX4-U46C (tga>tgc)-IRES-puro	This paper	N/A
Plasmid: p442-FSH-mGPX4-IRES-puro	Ingold et al.^21^	N/A
Plasmid: p442-FSH-mGPX4-U46C-IRES-puro	Ingold et al.^21^	N/A
Plasmid:141-codon optimized hFSP1 (NP_001185625.1)-Strep-IRES-puro	This paper	N/A

**Software and algorithms**		

GraphPad Prism v10	GraphPad Software	https://www.graphpad.com
ImageJ/Fiji software (v 1.53)	NIH	https://imagej.net/software/fiji/downloads

### Resource availability

#### Lead contact

Further information and requests for resources and reagents should be directed to and will be fulfilled by the lead contact, Marcus Conrad (marcus.conrad@helmholtz-munich.de)

#### Materials availability

All reagents and materials are listed in key resource table. Materials are available on reasonable request.

#### Data and code availability

• All data is provided in supplementary files.
• This paper does not report original code.
• Any further information needed to re-analyze the data reported in this paper is available from the lead contact upon request.

### Experimental model and subject details

#### Cell lines

4-OH-tamoxifen (Tam)-inducible *Gpx4*^−/−^ murine immortalized fibroblasts derived from a male embryo (referred to as Pfa1) were reported previously.^19^ HEK293T (CRL-3216), HT-1080 (CCL-121) and A375 (CRL-1619) cells were obtained from ATCC. Cells were cultured in DMEM-high glucose (4.5 g glucose/L) supplemented with 10% fetal bovine serum, 2 mM L-glutamine and 1% penicillin/streptomycin. *GPX4* knockout (KO) cells were cultured in medium containing 1 μM liproxstatin-1 to prevent ferroptosis. All cells were cultured at 37°C with 5% CO_2_ and verified to be negative for mycoplasma.

### Method details

#### Preparation of whole cell lysates and tagged GPX4

To prepare whole cell lysates, WT and *GPX4* KO cells were harvested and lysed by incubating protein lysates with lysis buffer (50 mM Tris-HCl pH 7.5, 300 mM NaCl, 1 mM dithiothreitol [DTT] and 0.1% NP-40) containing protease and phosphatase inhibitor cocktail (cOmplete and phoSTOP; Roche, Cat#04693116001 and Cat#4906837001) on ice for 30 min. Cell lysates were centrifuged at 20,000 x g for 30 min at 4°C and supernatant was collected.

To isolate tagged GPX4, HEK293T cells (approximately 3.6 x 10^6^ cells/dish) were seeded on 10 cm dishes and incubated overnight. When cells became 60–70% confluent, they were transfected with the 141-Strep-tagged human GPX4 plasmids or p442-Strep-tagged mouse GPX4 plasmids^21^ using PEI MAX (Polysciences, Cat#24765). To promote the expression level of selenoproteins including GPX4, 100 nM sodium selenite was supplemented to the medium.^33^ Forty-eight to 72 h after the transfection, the cells were harvested and lysed in lysis buffer on ice for 30 min. Cell lysates were centrifuged at 20,000 x g for 30 min at 4°C to remove cell debris. To isolate Strep-GPX4, the supernatant was incubated with MagStrep XT beads (IBA Lifesciences, Cat#2-4090-002) at 4°C for 1 h on a rotator. The beads were washed three times with washing buffer (100 mM Tris-HCl pH 8, 150 mM NaCl and 1 mM EDTA) followed by elution using the elution buffer (100 mM Tris-HCl pH 8, 150 mM NaCl, 1 mM EDTA and 50 mM biotin).

Protein concentrations of whole cell lysates and isolated GPX4 were measured by the BCA assay or determined by the coefficient using ExPASy ProtParam (https://web.expasy.org/protparam/) based on the absorbance value at 280 nm measured by UV5Nano spectrophotometer (Mettler Toledo).

#### Preparation of phospholipid hydroperoxides

Pure PCOOH (16:0/18:2-PCOOH) was prepared as previously mentioned.^20^ Pure 16:0/20:4-PCOOH and 16:0/18:2 PEOOH were prepared by photo-oxidation of 1-palmitoyl-2-arachidonoyl-*sn*-glycero-3-phosphocholine (16:0/20:4 PC, Avanti 850459C) and 1-stearoyl-2-linoleoyl-*sn*-glycero-3-phosphoethanolamine (16:0/18:2 PE, Avanti 850802C) respectively, according to previous reports with minor modifications.^34^^,^^35^ Crude PCOOH was prepared by photo-oxidation of soybean PC (Sigma Aldrich, Cat#P7443) according to previous reports with minor modifications.^34^^,^^35^ Briefly, PC was dissolved in 10 mL of methanol containing 10 μM rose engal. Then, the sample was placed under the LED light and photo-oxidized for 24 h at 4°C. To remove rose engal, the sample was subsequently passed through a Sep-Pak QMA cartridge (360 mg, Waters) followed by evaporation and reconstitution in ethanol.

#### Measurement of GPX4 activity

The enzymatic reaction was performed in 384 wells (total volume 50 μL/well) or 96 wells plates (total volume 100 μL/well) according to a previous report with minor modifications.^23^ For measurement of GPX4 activity, the assay buffer (100 mM Tris/Base, pH 8.0 containing 1.5 mM sodium azide, 2 mM EDTA, 0.1% Triton X-100, 1.5 IU/mL glutathione reductase and 1 mM GSH) and samples (isolated GPX4 enzymes [3–30 ng/well] or whole cell lysates [30 μg]) was first added to the well. Next, the mixture (in total, 10 μL/well) of NADPH (final concentrations, 500 μM) and purified PCOOH (final concentrations, 100 μM) was simultaneously added to the well immediately before starting the measurement. The consumption of NADPH was kinetically monitored by the absorption of 340 nm every 30 s at 37°C using a SpectraMax M5 or iD5 microplate reader with SoftMax Pro v.7 (Molecular Devices). In Figure 4E, crude oxidized PC (10 mg/mL, final 0.5 μg/μL), H_2_O_2_ (100 μM), *tert*-butyl hydroperoxide (100 μM) and cumene hydroperoxide (100 μM) were used as a substrate instead of purified PCOOH.

The percent of remaining NADPH was calculated as follows:

(Each sample of absorption (time = x)/the absorption (t = 0)) x 100

The GPX4 activity was estimated by NADPH consumption:

Remaining NADPH [substrate (−)] – remaining NADPH [substrate (+)]

#### Pre-treatment with GPX4 inhibitors

Strep-GPX4 was isolated from HEK293T cells overexpressing Strep-hGPX4 pre-treated with or without 10 μM RSL3 for 30 min at 37°C. For measurement of GPX4 activity, first, assay buffer (100 mM Tris/Base, pH 8.0 containing 1.5 mM sodium azide, 2 mM EDTA, 0.1% Triton X-100, 1.5 IU/mL glutathione reductase and 1 mM GSH) and samples (isolated GPX4 enzymes [3 ng/well]) were added to the well. Next, the mixture (in total, 10 μL/well) of NADPH (final concentrations, 500 μM) and purified PCOOH (final concentrations, 100 μM) was simultaneously added to the well immediately before starting the measurement.

#### Post-incubation with GPX4 inhibitors

Following affinity purification of Strep-hGPX4, isolated hGPX4 (3 ng/well of Strep-hGPX4) was incubated with 0.1–10 μM RSL3 or DMSO (equivalent volume of inhibitors) for 30 min at 37°C. For measurement of GPX4 activity, first, assay buffer (100 mM Tris/Base, pH 8.0 containing 1.5 mM sodium azide, 2 mM EDTA, 0.1% Triton X-100, 1.5 IU/mL glutathione reductase and 1 mM GSH) and samples (isolated hGPX4 enzymes [3 ng/well] post-incubated with GPX4 inhibitors) were added to the well. Next, the mixture (in total, 10 μL/well) of NADPH (final concentrations, 500 μM) and purified PCOOH (final concentrations, 100 μM) was simultaneously added to the well immediately before starting the measurement.

#### Immunoblotting and Coomassie Brilliant Blue staining

Cells were lysed in lysis buffer supplemented with protease and phosphatase inhibitor cocktail (cOmplete and phoSTOP), and centrifuged at 20,000 x g, 4°C for 30 min. The supernatants were sampled by adding 6 x SDS sample buffer (375 mM Tris-HCl, pH 6.8, 9% SDS, 50% glycerol, 9% β-mercaptoethanol, 0.03% bromophenol blue). After heating at 98°C for 3 min, the samples were resolved on 12% SDS-PAGE gels and subsequently electroblotted onto a PVDF membrane (Bio-Rad, Cat#170–4156). The membrane was blocked with 5% skim milk (Carl Roth, Cat#T145.2) in TBS-T (20 mM Tris-HCl, 150 mM NaCl and 0.1% Tween 20), then probed with the primary antibodies against GPX4 (1:1000, Abcam, Cat#ab125066), FSP1 (1:1000, Santa cruz, Cat#sc-377120, AMID), horseradish-peroxidase-conjugated β-actin (1:50,000, Sigma Aldrich, Cat#A3854) diluted in 5% skim milk in TBS-T overnight. After washing with TBS-T, the secondary antibody (1:3000, Cell Signaling, Cat#7074S for rabbit, Cat#7076S for mouse) diluted in 5% skim milk in TBS-T were incubated for 1 h and, antibody-antigen complexes were detected by the ChemiDoc Imaging System with Image Lab v6.0 (BioRad). Representative images are shown after the adjustment to the appropriate brightness and angle using the ImageJ/Fiji software (v 1.53).

For Coomassie Brilliant Blue (CBB) staining, the SDS-PAGE gel was stained with Coomassie staining solution (1 mg/mL Coomassie Brilliant Blue G-250 [Sigma Aldrich, Cat#115444002], 50% methanol and 10% acetic acid) for 15 min and subsequently soaked in washing buffer (70% methanol and 7% acetic acid) until the protein bands gave clear signals.

#### Generation of GPX4 KO cells

Genomic *Gpx4* deletion in Pfa1 cells was achieved by Tam-inducible Cre recombinase using the CreERT2/*LoxP* system.^19^ After 4-hydroxy tamoxifen treatment, a single clone of *Gpx4* ^−/−^ Pfa1 cells was isolated. HT-1080 and A375 *GPX4* knockout cells were established by transient co-transfection of two Cas9/sgRNA expressing vectors (lentiCRISPR-v2 puro and blast, Addgene: Cat#52961 and Cat#83480, respectively) with inserted the guide RNA sequence against *hGPX4* (5′-caccGCGTGTGCATCGTCACCAACG (for puro vector) and 5′-caccGCACGCCCGATACGCTGAGTG (for blast vector)). After antibiotic selection, single-cell cloning was performed as previously described.^7^^,^^10^

#### Generation of GPX4 overexpressing vectors

p442-Strep-tagged mouse GPX4^WT^ and GPX4^U46C^ plasmids containing the endogenous selenocysteine insertion sequence of *GPX4* were used as expressing vector.^21^ 141-Strep tagged-human GPX4^WT^ and GPX4^U46C^ expression plasmids were generated by subcloning from the sequence of p442-human GPX4-blast vector (short form of GPX4, GenBank: NM_001367832.1)^7^ using PCR and followed by the seamless cloning with In-Fusion enzyme (Takara, Cat#638948) and *EcoR*I digested plasmids. Human *GPX4* U46C (tga>tgc) mutant was generated by PCR with the following primers (forward: 5′-aacgtggcctcccagtgCggcaagaccgaagta-3′, and reverse: 5′-tacttcggtcttgccGcactgggaggccacgtt-3′).^36^ The final sequence of the insert into the plasmid was confirmed by Sanger sequencing as follows; the amino acid sequence is described in Figure S1.

#### hGPX4^WT^

gaattcgccgccaccATGGATTATAAAGATGATGATGATAAAGGGTCGGCCGCCGCCTGGAGCCACCCTCAGTTCGAGAAGGGAGGAGGAAGCGGCGGAGGCAGCGGAGGAGGAAGCTGGAGCCACCCGCAGTTCGAGAAAGGAGCTAGCTACCCATACGATGTTCCAGATTACGCTTGCGCGTCCCGGGACGACTGGCGCTGTGCGCGCTCCATGCACGAGTTTTCCGCCAAGGACATCGACGGGCACATGGTTAACCTGGACAAGTACCGGGGCTTCGTGTGCATCGTCACCAACGTGGCCTCCCAGTGAGGCAAGACCGAAGTAAACTACACTCAGCTCGTCGACCTGCACGCCCGATACGCTGAGTGTGGTTTGCGGATCCTGGCCTTCCCGTGTAACCAGTTCGGGAAGCAGGAGCCAGGGAGTAACGAAGAGATCAAAGAGTTCGCCGCGGGCTACAACGTCAAATTCGATATGTTCAGCAAGATCTGCGTGAACGGGGACGACGCCCACCCGCTGTGGAAGTGGATGAAGATCCAACCCAAGGGCAAGGGCATCCTGGGAAATGCCATCAAGTGGAACTTCACCAAGTTCCTCATCGACAAGAACGGCTGCGTGGTGAAGCGCTACGGACCCATGGAGGAGCCCCTGGTGATAGAGAAGGACCTGCCCCACTATTTCTAGCTCCACAAGTGTGTGGCCCCGCCCGAGCCCCTGCCCACGCCCTTGGAGCCTTCCACCGGCACTCATGACGGCCTGCCTGCAAACCTGCTGGTGGGGCAGACCCGAAAATCCAGCGTGCACCCCGCCGGAGGAAGGTCCCATGGCCTGCTGGGCTTGGCTCGGCGCCCCCACCCCTGGCTACCTTGTGGGAATAAACAGACAAATTAGgaattc.

#### hGPX4^U46C^

gaattcgccgccaccATGGATTATAAAGATGATGATGATAAAGGGTCGGCCGCCGCCTGGAGCCACCCTCAGTTCGAGAAGGGAGGAGGAAGCGGCGGAGGCAGCGGAGGAGGAAGCTGGAGCCACCCGCAGTTCGAGAAAGGAGCTAGCTACCCATACGATGTTCCAGATTACGCTTGCGCGTCCCGGGACGACTGGCGCTGTGCGCGCTCCATGCACGAGTTTTCCGCCAAGGACATCGACGGGCACATGGTTAACCTGGACAAGTACCGGGGCTTCGTGTGCATCGTCACCAACGTGGCCTCCCAGTGCGGCAAGACCGAAGTAAACTACACTCAGCTCGTCGACCTGCACGCCCGATACGCTGAGTGTGGTTTGCGGATCCTGGCCTTCCCGTGTAACCAGTTCGGGAAGCAGGAGCCAGGGAGTAACGAAGAGATCAAAGAGTTCGCCGCGGGCTACAACGTCAAATTCGATATGTTCAGCAAGATCTGCGTGAACGGGGACGACGCCCACCCGCTGTGGAAGTGGATGAAGATCCAACCCAAGGGCAAGGGCATCCTGGGAAATGCCATCAAGTGGAACTTCACCAAGTTCCTCATCGACAAGAACGGCTGCGTGGTGAAGCGCTACGGACCCATGGAGGAGCCCCTGGTGATAGAGAAGGACCTGCCCCACTATTTCTAGCTCCACAAGTGTGTGGCCCCGCCCGAGCCCCTGCCCACGCCCTTGGAGCCTTCCACCGGCACTCATGACGGCCTGCCTGCAAACCTGCTGGTGGGGCAGACCCGAAAATCCAGCGTGCACCCCGCCGGAGGAAGGTCCCATGGCCTGCTGGGCTTGGCTCGGCGCCCCCACCCCTGGCTACCTTGTGGGAATAAACAGACAAATTAGgaattc.

#### Measurement of PCOOH using LC-MS/MS

In Figures 2D and 3G, hGPX4^WT^ and hGPX4^U46C^ were isolated from HEK293T cells overexpressing hGPX4^WT^ and hGPX4^U46C^, respectively. In Figure 4D, hGPX4 was isolated from HEK293T cells overexpressing hGPX4^WT^ treated with or without RSL3 (10 μM, 30 min). Subsequently, isolated hGPX4 (30 ng per tube) were mixed with 1 mM GSH and 100 μM PCOOH for 30 min at 37°C. Then, 5 μL of the mixture was sampled by mixing with 495 μL of methanol. In Figure 4H, isolated hGPX4^WT^ was incubated with 1 μM RSL3 or DMSO for 30 min at 37°C in a 1.5 mL tube. Then, RSL3-treated hGPX4 was incubated with 1 mM GSH and 100 μM PCOOH for 30 min at 37°C. After the incubation, 5 μL of the mixture was sampled by mixing with 495 μL of methanol.

These methanol samples were centrifugated and the supernatant was subjected to an LC-MS/MS system consisting of a 7500 QTRAP tandem mass spectrometer (SCIEX) equipped with an Exion LC system (SCIEX). Chromatographic separation was performed using an Inertsustain AQ-C18 (3 μm, 2.1 × 150 mm; GL Science) at 40°C. The column was eluted with a mobile phase consisting of solvent A (water) and solvent B (methanol). The mobile phase gradient profile was as follows: 0–2 min, 95% B; 2–10 min, 95–100% B linear; 10.1 min, 100% B. The flow rate was 0.4 mL/min. The general LC-MS/MS conditions were as follows: entrance potential, 14.0 V; collision energy, 58.0 V; collision cell exit potential, 30.0 V; temperature, 500°C; source, ESI; and ion polarity, positive. PCOOH (1-palmitoyl-2-hydroperoxyoctadecadienoyl-*sn*-glycero-3-phosphocholine) was detected by multiple reaction monitoring (MRM) for the transition of precursor ions to products: (*m/z* 812 > 541).^20^^,^^37^

#### LOPAC library screen for FSP1 inhibitors

Pfa1 and Pfa1 *Gpx4* KO cells stably overexpressing human FSP1 were seeded on 96-well plates (2000 cells per well) and screened with a LOPAC library in principle as described.^9^ The viability of the different cell lines was assessed 48 h after treatment using AquaBluer. Compounds showing selective lethality in Pfa1 *Gpx4* ^KO^ cells stably overexpressing human FSP1 were validated in cell viability and *in vitro* FSP1 enzymatic assays, as described below.

#### Cell cytotoxicity measurement

Cells were seeded on 96-well plates (3,000 cells/well) and cultured overnight. The next day, the medium was changed, and the compounds were added at the indicated concentrations. Cell viability was determined after 24 h upon treatment, using 0.004% resazurin as an indicator of viable cells. As readout, fluorescence was measured at Ex/Em = 540/590 nm using a SpectraMax iD5 microplate reader with SoftMax Pro v7 (Molecular devices) after 4 h of incubation in the normal cell-culture medium. Cell viability (%) was normalized and calculated using untreated conditions.

For the LDH release assay, 3,000 cells/well were seeded on 96-well plates and cultured overnight. On the next day, the medium was changed to the fresh DMEM containing inhibitors and incubated for another 24 h. Necrotic cell death was determined using the Cytotoxicity Detection kit (LDH) following the manufacturer’s protocol (Roche, Cat#11644793001). In brief, cell-culture supernatant was collected as a sample of the medium, and cells were then lysed with 0.1% Triton X-100 in PBS as a lysate sample. Medium and lysate samples were individually mixed with reagents on the 96-well plate, and the reaction mixture was incubated for 15–30 min at room temperature. Then, the absorbance was measured at 492 nm using the SpectraMax iD5 microplate reader (Molecular Devices). The necrotic cell death ratio was calculated by LDH release (%) as follows: (absorbance (abs) of medium sample)/((abs of lysate) + (abs of medium samples)) × 100.

#### Preparation of recombinant hFSP1

Recombinant human FSP1 enzyme was produced in BL21 *Escherichia coli* and purified by affinity chromatography with a Ni-NTA system as described previously.^11^

#### Preparation of tagged FSP1

To generate tagged FSP1, HEK293T cells (approximately 3.6 x 10^6^ cells/dish) were seeded on 10 cm dishes and incubated overnight. When cells became 60–70% confluent, they were transfected with the 141-Strep-tagged human FSP1 plasmids using PEI MAX (Polysciences, Cat#24765). Seventy-two h after the transfection, the cells were harvested and lysed in lysis buffer on ice for 30 min. Cell lysates were centrifuged at 20,000 x g for 30 min at 4°C. To isolate strep-tagged hFSP1, the collected supernatant was incubated with MagStrep XT beads (IBA Lifesciences, 2-4090-002) at 4°C for 1 h on a rotator. The beads were washed three times with washing buffer (100 mM Tris-HCl pH 8, 150 mM NaCl and 1 mM EDTA) followed by elution using the elution buffer (100 mM Tris-HCl pH 8, 150 mM NaCl, 1 mM EDTA 50 mM biotin and 1 mM DTT). Protein concentration of the whole cell lysate and isolated FSP1 was measured by the coefficient using ExPASy ProtParam (https://web.expasy.org/protparam/) based on the absorbance value at 280 nm measured by UV5Nano spectrophotometer (Mettler Toledo).

#### FSP1 enzyme assay

For the resazurin-based FSP1 activity assay, reaction solutions in TBS buffer (50 mM Tris-HCl, 150 mM NaCl) containing 25–200 nM isolated Strep-hFSP1 or 50 nM recombinant hFSP1, 200 μM NADH or 200 μM NADPH and the inhibitors (iFSP1 and WIN62577) were prepared. After the addition of 100 μM resazurin sodium salt, the fluorescence intensity (FL intensity, excitation/emission wavelengths (Ex/Em) = 540 nm/590 nm) was monitored every 60 s at 37°C using SpectraMax M5 or iD5 microplate reader with SoftMax Pro v7 (Molecular devices). Reactions without inhibitors were used to normalize and calculate FSP1 enzymatic activity and IC_50_ values. Curve fitting and calculation of IC_50_ values were performed using GraphPad Prism v10.

### Quantification and Statistical analysis

Statistical information for individual experiments can be found in the corresponding figure legends. Graphs were created using GraphPad Prism v10 (GraphPad Software).

## Supplemental information

Document S1. Figures S1 and S2

Document S2. Article plus supplemental information
