# Supplementary material for: A tangible method to assess native ferroptosis suppressor activity
Source: Cell Rep Methods. 2024 Feb 24;4(3):100710. doi: 10.1016/j.crmeth.2024.100710 (PMC10985226; doi:10.1016/j.crmeth.2024.100710)
Supplement: Document S1. Figures S1 and S2 [file mmc1.pdf]

Cell Reports Methods, Volume *4*

Supplemental information

A tangible method to assess native ferroptosis suppressor activity

Toshitaka Nakamura, Junya Ito, André Santos Dias Mourão, Adam Wahida, Kiyotaka Nakagawa, Eikan Mishima, and Marcus Conrad

Figure S1

a **DNA (*hGPX4^{WT}*, *hGPX4^{U46C}* (*TGA > TGC*))**

gaattcgccgccaccATGGATTATAAAGATGATGATGATAAAGGGTCGGCCGCCGCCTGGAGCCACCCTCAGTTCGAGAAG
GGAGGAGGAAGCGGCGGAGGCAGCGGAGGAGGAAGCTGGAGCCACCCGCAGTTCGAGAAAGGAGCTAGCTACC
CATACGATGTTCCAGATTACGCTTGCGCGTCCCGGGACGACTGGCGCTGTGCGCGCTCCATGCACGAGTTTTCCGC
CAAGGACATCGACGGGCACATGGTTAACCTGGACAAGTACCGGGGCTTCGTGTGCATCGTCACCAACGTGGCCTC
CCAGTGAGGCAAGACCGAAGTAAACTACACTCAGCTCGTCGACCTGCACGCCCGATACGCTGAGTGTGGTTTGCG
GATCCTGGCCTTCCCGTGTAACCAGTTCGGGAAGCAGGAGCCAGGGAGTAACGAAGAGATCAAAGAGTTCGCCGC
GGGCTACAACGTCAAATTCGATATGTTCAGCAAGATCTGCGTGAACGGGGACGACGCCCACCCGCTGTGGAAGTG
GATGAAGATCCAACCCAAGGGCAAGGGCATCCTGGGAAATGCCATCAAGTGGAACTTCACCAAGTTCCTCATCGAC
AAGAACGGCTGCGTGGTGAAGCGCTACGGACCCATGGAGGAGCCCCTGGTGATAGAGAAGGACCTGCCCCACTAT
TTCTAGCTCCACAAGTGTGTGGCCCCGCCCGAGCCCCTGCCCACGCCCTTGGAGCCTTCCACCGGCACTCATGAC
GGCCTGCCTGCAAACCTGCTGGTGGGGCAGACCCGAAAATCCAGCGTGCACCCCGCCGGAGGAAGGTCCCATGG
CCTGCTGGGCTTGGCTCGGCGCCCCCACCCCTGGCTACCTTGTGGGAATAAACAGACAAATTAGgaattc

Amino acids (hGPX4^{WT})

MDYKDDDDKGSAAAWSHPQFEKGGGSGGGSGGGSWSHPQFEKGASYPYDVPDYACASRDDWRCARSMHEFSA
KDIDGHMVNLDKYRGFVCIVTNVASQUGKTEVNYTQLVDLHARYAECGLRILAFPCNQFGKQEPGSNEEIKEFAAGY
NVKFDMFSKICVNGDDAHPLWKWMKIQPKGKGILGNAIKWNFTKFLIDKNGCVVKRYGPMEEPLVIEKDLPHYF*

Annotation

M – FLAG tag – G (linker) – twin-Strep tag – GAS(linker) – HA tag – short form GPX4 – U46
– STOP – SECIS (selenocysteine insertion sequence)

b **DNA (codon optimized hFSP1)**

gccgccaccatgggttctcaagtgtcagtggaatcaggggctctccacgttgtaattgtgggcgggggatttggcgggatagctgccgcatcccaacttcaggcattgaat
gtgccatttatgctggtcgacatgaaggacagctttcatcataatgtggctgccctcagagcctccgtggaaacaggcttcgcaaaaaaaacttttatatcctattccgtaac
attcaaggacaactttcgccaagggttggtagttgggattgacctgaagaatcaaatggtactcctgcagggaggcgaagctctgccgttcagccatctcatcctggctac
cggttctactggtccatttcctggcaagttcaacgaagtctcttcacaacaagcggccatacaagcctacgaggatatggtgcgccaggtccagagaagtaggtttattgta
gtggtagggggtggctcagccggtgtcgagatggctgcagagattaaaacagagtatcctgagaaggaagtcactctgatacactctcaagtggcactcgccgataaa
gagcttttgcctagtgtgagacaagaggtcaaggagatactgctcagaaaaggcgtccaacttctgctcagcgaacgagtttctaacctcgaggaactccctctcaacga
atatagggagtacattaaggtgcaaacggataagggaacggaagtggcgacaaacctcgttattctctgcactgggatcaaaattaattcttccgcataccggaaggcgt
tcgagtctcgactcgccagctccggagccctgcgcgtaaatgagcacctgcaagtggagggccattctaacgtctatgcgatcggagattgcgctgacgtccgcacgcct
aagatggcttaccttgctgggctgcatgcaaacattgcggtcgctaatatagttaacagtgttaaacaaaggccgctgcaagcgtataagcctggtgccctgacgttcttgct
gtctatgggtcgaaacgatggtgtagggcagataagtggattttacgtgggtcgattgatggtccgccttacgaagtctagggacctgtttgttagtacgagttggaagacga
tgagacaatcccccctggaggtgattacaaggatgacgacgataaagggtcagcatctgcctggagtcatccgcaatttgaaaaaggtgggggttccggcggcggta
gtggtggaggatcttggtctcacccccagttcgaaaaaggatag

Amino acids (hFSP1)

MGSQVSVESGALHVVIVGGGFGGIAAASQLQALNVPFMLVDMKDSFHHNVAALRASVETGFAKKTFISYSVTFKDNF
RQGLVVGIDLKNQMVLLQGGEALPFSHLILATGSTGPFPGKFNEVSSQQAAIQAYEDMVRQVQRSRFIVVVGGGSAG
VEMAAEIKTEYPEKEVTLIHSQVALADKELLPSVRQEVKEILLRKGVQLLLSERVSNLEELPLNEYREYIKVQTDKGTEV
ATNLVILCTGIKINSSAYRKAFESRLASSGALRVNEHLQVEGHSNVYAIGDCADVRTPKMAYLAGLHANIAVANIVNSV
KQRPLQAYKPGALTFLLSMGRNDGVGQISGFYVGRLMVRLTKSRDLFVSTSWKTMRQSPPGGDYKDDDDKGSASA
WSHPQFEKGGGSGGGSGGGSWSHPQFEKG*

Annotation

hFSP1 – GG (linker) – FLAG tag – GSA (linker) – twin-Strep tag – STOP

Figure S1. | DNA and protein sequence for GPX4 and FSP1, related to Figure 2/5 and STAR Methods.
a. Schematic representation of the human GPX4 construct (DNA and protein) used in this study.
b. Schematic representation of the human FSP1 construct (DNA and protein) used in this study.

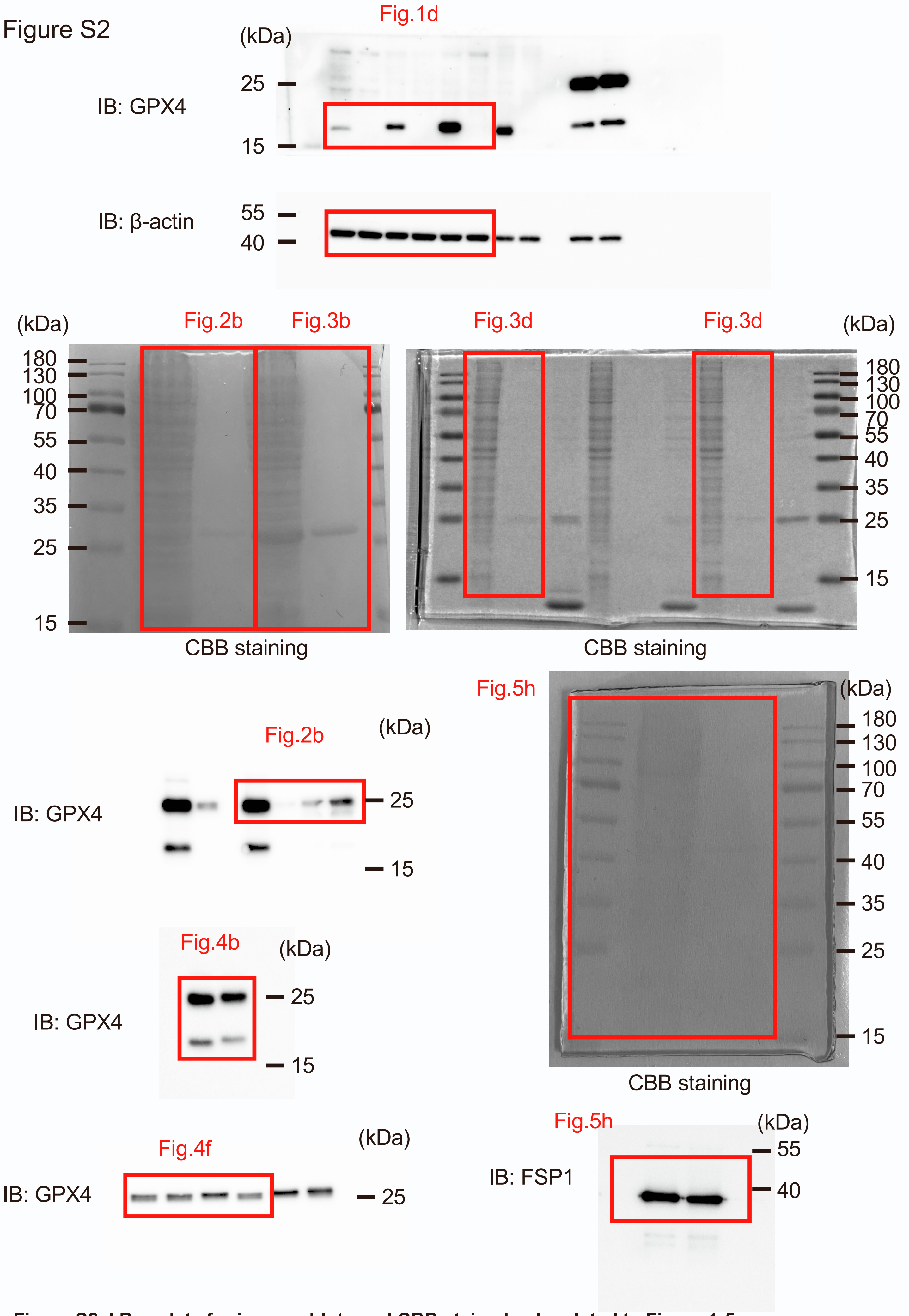

Figure S2. | Raw data for immunoblots and CBB stained gels related to Figure 1-5.
Original raw immunoblotting and CBB staining gel images with a molecular marker were shown.
Red areas were cropped for visualization and corresponding to the indicated main figure panels.
